# The Potential Role of Exosomes in the Treatment of Brain Tumors, Recent Updates and Advances

**DOI:** 10.3389/fonc.2022.869929

**Published:** 2022-03-17

**Authors:** Zoufang Huang, Shayan Keramat, Mehrdad Izadirad, Zhe-Sheng Chen, Mohammad Soukhtanloo

**Affiliations:** ^1^ Ganzhou Key Laboratory of Hematology, Department of Hematology, The First Affiliated Hospital of Gannan Medical University, Ganzhou, China; ^2^ Department of Hematology and Blood Bank, Faculty of Medicine, Mashhad University of Medical Science, Mashhad, Iran; ^3^ Department of Hematology and Blood Bank, School of Allied Medical Sciences, Shahid Beheshti University of Medical Sciences, Tehran, Iran; ^4^ Department of Pharmaceutical Sciences, St John’s University, New York, NY, United States; ^5^ Department of Clinical Biochemistry, Faculty of Medicine, Mashhad University of Medical Sciences, Mashhad, Iran; ^6^ Pharmacological Research Center of Medicinal Plants, Mashhad University of Medical Sciences, Mashhad, Iran

**Keywords:** exosomes, extracellular vesicles, brain tumor, tumor environment, blood-brain barrier, treatment

## Abstract

Exosomes are small endosomal derived membrane extracellular vesicles that contain cell-specific cargos such as lipid, protein, DNA, RNA, miRNA, long non-coding RNA, and some other cell components that are released into surrounding body fluids upon the fusion of multivesicular bodies (MVB) and the plasma membrane. Exosomes are a one-of-a-kind cell-to-cell communication mechanism that might pave the way for target therapy. The use of exosomes as a therapeutic potential in a variety of cancers has been and is still being investigated. One of the most important of these has been the use of exosomes in brain tumors therapy. Exosome contents play a crucial role in brain tumor progression by providing a favorable niche for tumor cell proliferation. Also, exosomes that are secreted from tumor cells, lead to the protection of tumor cells and their proliferation in the tumor environment by reducing the inflammatory response and suppression of the immune system. Although some treatment protocols such as surgery, chemotherapy, and radiotherapy are common in brain tumors, they do not result in complete remission in the treatment of some malignant and metastatic brain tumors. Identifying, targeting, and blocking exosomes involved in the progression of brain tumors could be a promising way to reduce brain tumor progression. On the other way, brain tumor therapy with effective therapeutic components such as siRNAs, mRNAs, proteins, could be developed. Finally, our research suggested that exosomes of nanoscale sizes might be a useful tool for crossing the blood-brain barrier and delivering effective content. However, further research is needed to fully comprehend the potential involvement of the exosome in brain tumor therapy protocols.

## Introduction

Exosomes are small endosomal derived membrane microvesicles that contain cell-specific cargos such as lipid, protein, DNA, RNA, miRNA, long non-coding RNA, and some other cell components that are released into surrounding body fluids upon the fusion of multivesicular bodies (MVB) and the plasma membrane (1).

In the 1980s, researchers discovered the presence of some structure like a tiny bubble in the extracellular space. Initially, considered as cellular waste resulting from cell damage and has no significant impact on neighboring cells (2). Actually, with the advancement of technology and further studies, they found that it seems the cells in the neighborhood of each other released these bubble-like bodies that are now known as exosomes, to transfer messages between themselves. Gradually, it became clear that these messages were targeting cells farther away from their surroundings (3). Therefore, it is assumed that exosomes represent a novel mode of cell-to-cell communication, and this may show an important role of exosomes in many cellular interactions such as signal transduction and activation of some signaling pathways in recipient cells (4). The role of the vesicles is defined by exosomes composition include of death or survival or proliferation and differentiation, sharing of immune responses, immune modulators, antigen presentation, and some biological response (5).

Thus, exosomes could have a potential role in insights into target therapy. It has been indicated that the composition of exosomes can affect pharmacokinetic properties (6). Exosomes have the potential to aid in the prognosis and diagnosis of illnesses such as cancer, chronic inflammation, cardiovascular diseases, infections, and autoimmune, in addition to its therapeutic potential (7).

Because exosomes are released from many different cell types, including dendritic cells, macrophages, B cells, T cells, epithelial cells, platelets, mast cells, adipocytes, and fibroblasts, it is revealed that nervous system cells, including Schwann cells, astrocytes, and neurons also have a potential role to release exosomes (8, 9). Moreover, it has been demonstrated that exosomes are involved in the function of the nervous system, including the regulation of synaptic communication and nerve regeneration (10). Therefore, in recent times, exosomes in the nervous system are considered as a new bridge for intercellular communication that in addition to participation in normal neuronal physiology, also has an important role in the pathogenic event such as neurodegenerative disease as a pathogen transmitter or as therapeutic potential (11).

The use of exosomes as a therapeutic potential in many cancers has been and is still being investigated. One of the most important of these has been the use of exosomes in the treatment of brain tumors (12, 13).

Up to now, various treatments for brain tumors have been proposed, including surgery, radiotherapy, and chemotherapy. On the other hand, scientists have raised several concerns regarding many side effects and challenges in these types of treatments (14).

Maybe one of the most important challenges is the sensitivity of the brain tissue and the need for high precision in surgery, and on the other hand, the existence of a blood-brain barrier (BBB) to prevent drug treatments from entering the brain environment (15). However, due to the size of the exosome (30–150 nm), it is capable of easily crossing this barrier and pursuing therapeutic aims.

The relevance of these vesicles in brain malignancies, treatments, and pathophysiology will be discussed in this review (16).

## Exosome Biogenesis

**Figure 1:**
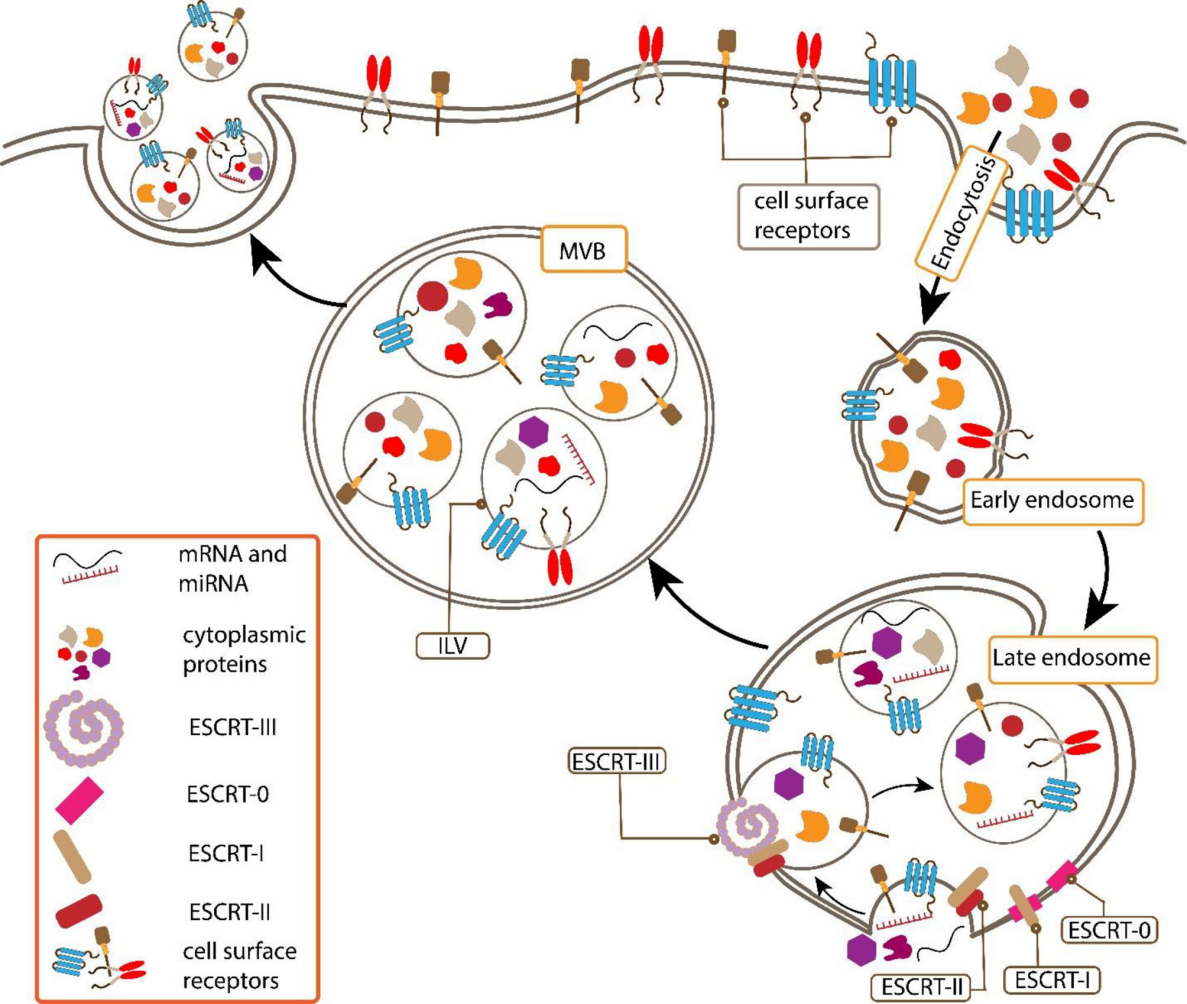
Early endosomes mature into late endosomes and exosomes are formed by inward budding of the multivesicular body (MVB) membrane. Intraluminal vesicles (ILVs) are formed by the Invagination of late endosomal membranes within large MVBs. It has been demonstrated that the endosomal sorting complex required for transport (ESCRT) machinery is important in this process.

The endosomal system has an important role in the biogenesis of exosomes. Early endosomes mature into late endosomes and exosomes formed by inward budding of the multivesicular body (MVB) membrane. Intraluminal vesicles (ILVs) are formed by the invagination of late endosomal membranes within large MVBs (17). It has been demonstrated that the endosomal sorting complex required for transport (ESCRT) machinery is important in this process. ESCRT includes four different protein complexes numbered ESCRT‐0, ESCRT‐I, ESCRT‐II, and ESCRT‐III, inside of some associated proteins such as AAA ATPase Vps4, TSG101, and ALIX (18, 19). Each of these protein components has been shown to have specific functions. ESCRT‐0 has an important role in the internalization of ubiquitinated proteins and also, sequestration of these proteins to particular domains of the endosomal membrane. Subsequently, ESCRT-I and ESCRT-II promote the budding process, which will then combine with ESCRT-III, and the final stage of membrane invagination and separation with the total complex of ESCRT is completed. Finally, these processes lead to multivesicular body (MVB) formation (19) (**Figure 1**).

Also, ALIX as an exosomal protein participates in the budding process and the corporation with syndecan has an important role in exosomal cargo selection. In addition, it has been shown that ALIX has an important role in changing the protein composition and also, secretion of exosomes (20). Also, it is demonstrated that ESCRT inhibition can lead to inhibiting the secretion of exosomes, also, in addition to exosome biogenesis ESCRT has another key role in cells’ biological function especially cytokinesis (18, 21). On the other, TSG101, ALIX, VPS4 proteins have been implicated that have a role in promoting the budding of exosome-like vesicles in T lymphocytes (22).

One of the most recent findings in the exosome biogenesis suggested an alternative pathway so-called “ESCRT-independent mechanisms”. It is demonstrated that this mechanism depends on raft-based microdomains and cargo loading involves lipids, sphingosine, hydrophobic modifications, and associated protein such as tetraspanins on rafted membranes (23). The presence of ceramide and sphingolipids molecules generates from ceramide by ceramidase and sphingosine kinase have an important role in the budding process of ILVs formation (24). Also, tetraspanins, which are proteins that organize membrane microdomains which are so-called tetraspanin‐enriched microdomains (TEMs), induce the ILVs formation in ESCRT‐independent mechanism by CD63 (a member of the tetraspanin family) function (23).

Phospholipase D2 (PLD2) is an important enzyme in the lipid modification process, has been shown to play an important role in exosome biogenesis as well as ILV formation. Phospholipase D2, by hydrolysis of phosphatidylcholine to phosphatidic acid (PA), induces a negative membrane curvature, which in turn leads to membrane invagination (25).

Another enzyme, called diacylglycerol kinase α (DGKα), that has a role in phosphate group insertion to the diacylglycerol (DAG), is also involved in the production of phosphatidic acid, which is participated in the release of exosomes (26).

Although, the biogenesis of exosomes has been introduced in two major pathways as ESCRT-dependent or ESCRT-independent mechanisms with different types of exosomes and different types of machinery such as ESCRT machinery, lipids, tetraspanins, PLD2, DGK, and other proteins (20–26). But in fact, exosome biogenesis can be the consequence of these two mechanisms Collaboration together. However, although there are many studies related to exosome biogenesis, it still seems that this issue needs further investigation because there are many unknown Pathways.

## Applications of Exosomes in Cancer Diagnosis and Treatment

The atmosphere surrounding the research field of exosomes in cancer has dramatically changed in recent years but the use of exosomes in cancer diagnosis or therapy was not impressive (27). However, studies are raising in this field and conducted research on exosomes have shown that exosomes might serve as a new instrument in cancer diagnosis and treatment.

The findings have been revealed that tumor-derived exosomes (TDEs) include components that are distinctive biomarkers and might be used to diagnose tumors. For example, it has been investigated that GPC1+-circulating exosomes increment is a diagnosis factor in patients with pancreatic ductal carcinoma (PDAC) and colorectal cancer (CRC) (28). Investigations in this field regarding lung cancer reveal that circulating exosomes carrying CD151, CD171, and tetraspanin 8 can be detected in lung cancer patients (29). These exosomes could serve as a distinguishing marker to discriminate lung cancer patients from non-lung cancer patients (29).

Besides plasma and serum, other sources such as urine, saliva, cerebrospinal fluid (CSF), and milk can be used for exosome isolation. Studies show that these sources are easy to use for purifying exosomes that have diagnostic applications (30). For example, urine-derived exosomes containing ITGA3 and ITGB1 have been shown to promote metastasis in lung cancer patients when compared to early stage patients (31).. As a result, the use of these exosomes may be effective in predicting metastasis in lung cancer patients (32).. Additionally, it has been shown that saliva-derived vesicles such as exosomes in head and neck carcinoma (HNC) displayed biomarkers like CD44 and CD95L (33). Since, saliva is a more accessible sample than another source, finding these saliva-derived exosomes may be the easiest and fastest way to HNC detection (33).

Interestingly, exosomes can transfer regulator elements such as RNA, DNA, or proteins that affect treatment response assessment. For example, it has been revealed that exosomes could establish chemoresistance in cancer cells, like HER-2 displaying exosomes could neutralize HER-2 antibody (Trastuzumab) and lead to chemoresistance in breast cancer (34). Recently, there has been an increasing interest in exosome-derived lncRNAs as factors in the development of tumor chemoresistance (35). Evidence suggests that lncRNAs transferred by exosomes may contribute to chemoresistance in other cells. For example, Han M et al. demonstrated that exosomes, such as EVs, contain the lncRNA, AFAP1-AS1, which causes trastuzumab resistance in breast cancer patients (36).

There are interesting pieces of evidence regarding exosome applicants in cancer therapy. It seems that the extracellular vesicles exosome can be a promising designable platform for transferring specific content such as drugs, proteins, and regulator RNAs due to their endogenous origin, stability, biocompatibility, and other unique features (37, 38). O’Brien and colleagues showed that exosomes filled by miR-134 could reduce cellular migration and invasion, and enhanced sensitivity to anti-Hsp90 drugs (39). Exosomes also can be enriched by chemotherapeutic drugs and target cancer cells. Bellavia D and colleagues have posited that imatinib or BCR-ABL siRNA-loaded exosomes will be able to target CML cells and unload their cargo to target cells. Forasmuch as IL-3R expression increases on CML cells, they have used this feature for CML cells targeting *via* producing IL3-Lamp2b expressing exosomes. They reported that these exosomes successfully target cancer cells and imatinib is delivered. As a result, cancer cell proliferation is reduced (40).

## Exosomes in Brain Tumors

It has been found that exosomes play a key role in brain tumors that can be effective in tumor growth and progression (41). Exosomes secreted from tumor cells in the brain can create a small communication between a malignant cell and surrounding cells in different ways, such as the release of proteins, mRNAs, or other cellular components involved in cell malignancy (10, 41).

Currently, treatment of brain cancers has been a major challenge compared to cancers in other organs due to the presence of BBB and the complexity of the central nervous system (CNS) microenvironment (42). The therapeutic role of exosomes in various brain cancers such as glioblastoma, neuroblastoma, medulloblastoma, astrocytoma, gliosarcoma, and oligodendroglioma can be effective (15, 43). Although this effect can have different aspects, as it can be effective in the early diagnosis of brain cancer, inhibition of the spread of exosomes containing cancer cell information to other healthy cells, or invitro biogenesis of exosomes and its use to transfer the effective factors for treatment to the microenvironment of the brain (43).

To investigate the effect of exosomes in the treatment of brain cancers, the role of exosomes in the development of various brain cancers and their progression must first be studied in more detail.

### Role of Exosome in Primary Brain Tumor

#### Glioblastoma

Glioblastoma (GBM) is the most aggressive one among tumors of glial origin that rapidly grow and spread into nearby brain tissue and forms from cells called astrocytes that support nerve cells. The survival of patients has been reported usually between 10–15 months (44). Currently, complete remission of GMB is impossible, and actually, treatments slow the progression of cancer and reduce symptoms (45).

Exosomes have an important role in the progression and development of GBM. This communication is between cancer stem cells, tumor cells, microglia, and parenchymal cells, and especially immune cells (46). Although it is similar to a hypothesis, the exosome traces in the glioma progression were detected by purifying the exosome in culture media containing murine glioma. Also, these findings indicated that glioma exosomes contained a variety of proteins involved in tumor progression and surface markers (47). The most important of them are heat shock proteins (HSPs) such as HSPB5, HSP 60, 70, and (48). This finding suggests that the presence of inflammatory cytokines, including interleukin (IL)-1, IL-6, and TNF-α in interaction with HSPs, increases its release, which may contribute to tumor cell immortality and disease progression (49).

In total, it has been shown that exosomes in solid tumors can help tumor progression by suppressing the immune system (49). Also, in GBM, exosomes secreted by tumor cells modulate the immune system reaction by inducing the presence of M2 macrophages around the tumor, thus helping the tumor to progress and escape from the immune system (50). In addition, Annexin A2 that plays a significant role in invasion, metastasis, angiogenesis, and proliferation is one of the important markers that has been found on the surface of exosomes in GBM (51). Despite Annexin A2, CD44 is also present on the surface of exosomes in GBM that plays as a receptor for hyaluronic acid and it is implicated that CD44 is a marker for cell motility, tumor growth, angiogenesis, and cancer stem cell (52, 53). It should be noted, one of the most important challenges in the treatment of GBM is drug resistance (54). Interestingly, It was found that the proteins involved in resistance to treatment that has been named the DNA-dependent protein kinase catalytic subunit (PRKDC), which is involved in the repair of double-strand DNA against radiotherapy, can be transported through exosomes, it is assumed that the inhibition of which can play an important role in the treatment of GBM (51).

Also, miRNA expression was detected in CSF and plasma of GBM patients. These exosomes were containing different miRNA with different activities and roles (55). Regardless of the role and activity of miRNA in GBM patients, their detection can be used as an effective diagnostic marker. In this regard, miR-221 and miR-21 have been evaluated as biomarkers in CSF of GBM patients (56, 57). As well, GBM patients which have received antitumor vaccines, miR-320 and miR-574-3p have been detected in the plasma and have a diagnostic role as biomarkers (43).

#### Neuroblastoma

Neuroblastoma (NB), as its name implies, is a malignancy involving nerve cells in the immature stages. NB is a malignant and very progressive cancer that is often detected in children under 5 years of age and with higher rates in infants or fetuses (58). It’s important to pay attention to this disease because it is the second most common malignancy in children and also one of the most common malignant cancers of the nervous system (59). NB usually originates in the adrenal glands but progresses rapidly throughout the body, such as the bones, abdomen, neck, chest, and even under the skin (58). Although the etiology of this cancer is not known exactly, the main cause of this disease is considered to be genetic mutations that can be acquired and sporadic or even in rare cases inherited from parents (60). According to the staging set by the International Neuroblastoma Staging System (INSS), it has been shown that in advanced stages, the NB deviates from the localized form and can progress rapidly to other tissues. This rapid progression similar to a metastatic process can be effectively correlated with the activity of exosomes (61).

Despite the GBM, there are few exosome studies in the field of NB, and the role of exosomes in the progression of NB remains unknown. The studies that have been done in this field so far are often in the field of treatment resistance and providing a suitable environment for tumor development (62). MYCN-amplified (a proto-oncogene that is associated with poor prognosis in NB) NB cells have been shown to play an important role in tumor progression and development by secreting some exosomal miRNAs and result in changing the tumor environment in favor of tumor growth (63).

Also, it has been demonstrated that exosomes with miR-155 which transferred from monocytes/macrophages to NB cells, and miRNA-21 which has been secreted from NB cells in around of tumor environment have a key role in resistance to chemotherapy, a process that occurs through miR-21/TLR8-NF-κB/exosomal miR-155/TERF1 signaling pathway (64, 65). Therefore, it can be said that inhibition of these exosomes maybe help the patients with NB to be cured and lead to a favorable prognosis in NB patients (62).

One of the most recent findings regarding exosomes in NB is the study of exosomal hsa-miR199a-3p (66). It has been shown that upregulation of exosomal hsa-miR199a-3p can be associated with high-risk and poor prognosis of NB. Although hsa-miR199a-3p is generally found in other malignancies and has opposite effects in different cancers, in NB has a progressive effect and poor prognosis (66). The mechanism of the hsa-miR199a-3p function is such that it reduces the expression of an enzyme called NEDD4. This enzyme plays a very important role in suppressing tumor activity by interacting with Myc *via* ubiquitination and degradation of Myc protein and catalyzing PTEN mono-ubiquitination and regulating PTEN nuclear translocation (67). As a result, inhibition of exosomal hsa-miR199a-3p can play an effective role in improving NB. Also, detecting the increase in the level of exosomal hsa-miR199a-3p can be a biomarker to detect NB in ​​the early stages or to determine the prognosis (66, 67).

Recently, it has been demonstrated that a high level of exosomal miR-375 correlates with BM metastasis in NB patients (68). Therefore, exosomal miR-375 may be an important novel biomarker in detecting BM metastatic progression, and also may represent a novel potential target for NB patients with BM metastasis (68, 69).

### Role of Exosomes in Metastasis to the Brain

To date, although many advances have been made in the treatment of various cancers, from chemotherapy to radiotherapy, immunotherapy, and surgical skills, the focus of these therapies has been on anti-tumor or anti-cancer activities (70). The missing puzzle of therapeutic protocols, on the other hand, maybe therapy against anti-metastatic activities. In recent years, many studies have been conducted on the role of exosomes in tumor metastasis (71).

Metastatic brain tumors are one of the most common brain tumors that can be secondary to a variety of tumors in different tissues of the body. Therefore, these tumors are also called secondary tumors (72). In addition, metastatic brain tumors are important because they grow rapidly and have more destructive effects on brain tissue than primary tumors. On the other hand, multiple metastatic tumors may involve the brain at the same time (73). Regarding the risk of metastatic brain tumors, it can be said that most malignancies and systemic tumors have a risk of metastasis to the brain, but what is more common is that tumors of the breast, lung, renal, and colon increase the risk of metastasis to the brain (74, 75). It has been shown that more than half of people with metastatic brain tumors have a history of non-small cell lung cancer (76, 77). Also, this risk is about 30% for patients with a history of breast cancer (74). Exosomal microRNAs have a crucial role in the development of malignancies, as previously stated. As a result, more research into these topics might be worthwhile (78). For example, High expression of miR-451a and miR-4257 have been shown to be closely related to non-small cell lung cancer tumor progression and poor prognosis (79). miR-21 is also associated with the recurrence and progression of lung cancer (79, 80). Actually, high levels of exosomal miR-23a that has angiogenesis activity, are found in non-small cell lung cancer patients which can also be effective in metastasis to the brain (81, 82).. It has been demonstrated that cancer cells in breast cancers secrete a large amount of exosomal miR-122 (83). By affecting normal cells in pre-metastatic sites, miR-122 stops glucose uptake during this process, thus providing the energy needed for the unbridled proliferation of tumor cells and the development of metastasis (84). Therefore, its inhibition can play an essential role in preventing the metastasis and progression of tumor cells (84, 85). The cells identified as receptors for miR-122 were fibroblasts, brain astrocytes, and neurons, indicating the importance of exosomal miR-122 role in metastasis to the brain (85, 86).

Also, miR-105 is secreted from Breast cancer cells and affects endothelial cells. This effect can be such that it leads to the destruction of endothelial barriers, which enhances the metastasis process (77). It has also been shown that the destruction of endothelial barriers leads to the destruction of the BBB, indicating the effective role of exosomal miR-105 in metastasis to the brain (87).

**Figure 2:**
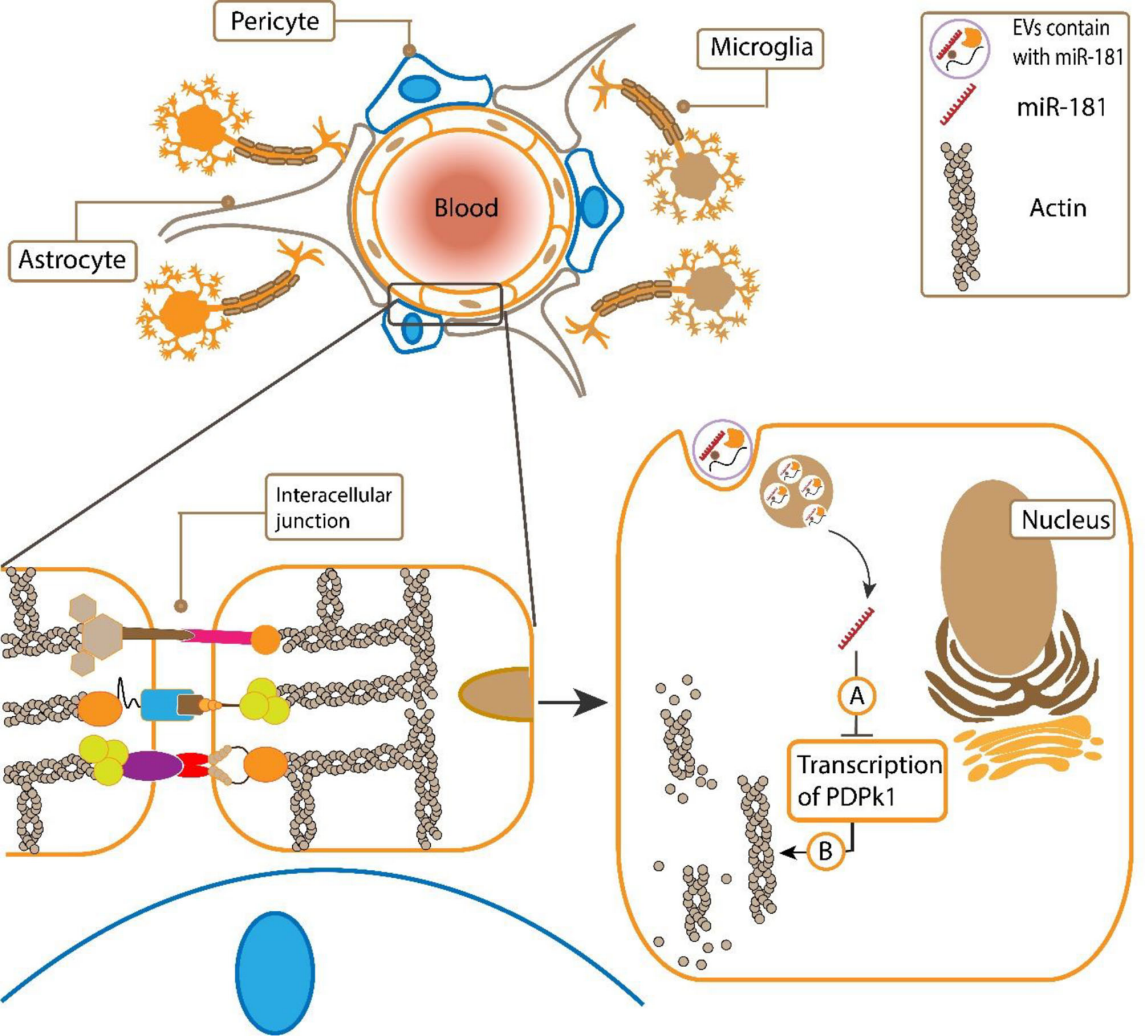
**(A)** miR-181c that secreted from metastatic breast cancer cells could transfer by exosomes to epithelial cells and suppresses the expression of PDPK1. **(B)** Then, it results in activated cofilin which disassembles actin filaments and makes the BBB permeable. Following, cancer cells could cross the BBB.

One of the most important microRNAs that recently have been identified and discussed in the study of metastasis between breast cancer cells and other tissues, especially the brains, is miR-181c (88). The direct effect of miR-181c on brain metastasis is due to the destruction of the BBB (81). The mechanism of degradation by miR-181c is by promoting the pathway of cofilin protein activation and finally by destroying actin filaments by it (81) (**Figure 2**).

In total, if we want to investigate other roles of exosomal contents (such as cytokines, enzymes, chemokines, etc.) in metastasis to the brain, we could consider their effective role in changing normal fibroblasts to tumor-associated fibroblasts and creating a suitable niche for the development of tumor cells (82, 89). Also, reducing the level of inflammation and escaping from the immune system, protects tumor cells and their proliferation in the tumor environment (90).

Further researches could yield novel insights into the molecular mechanisms of metastasis and the development of advanced therapeutic strategies to prevent the formation of brain metastasis.

## Conclusion

Brain tumors are important because, in addition to physical complications, they also have cognitive complications (91). To date, the treatment of brain tumors in medicine has been associated with many challenges. Surgery is still considered the most important step in treating brain tumors, but brain surgery is one of the most difficult types of surgery (92). In addition, the long recovery period after brain surgery and its complications should not be underestimated (92). Chemotherapy and radiotherapy are also prescribed for malignant tumors in the next step. Although Chemotherapy and radiotherapy have gradually become more effective with advances in medical science, it still does not bring complete remission in the treatment of some malignant and metastatic brain tumors. In addition, complications of Chemotherapy are also undeniable (93, 94).

In molecular studies of the pathogenesis of brain tumors, researchers focus on genetics and epigenetics, while cellular communications with each other and their surroundings play a very important role in tumor progression (95). Intercellular communications are formed by membrane extracellular vesicles, exosomes typically play a critical role in the transmission of biological processes between tumor cells and other cells and tissues (4, 96).

As mentioned, exosomes play an important role in the progression of brain tumors, and one of the most important roles of exosomes in brain tumors is in the metastasis of tumors to the brain, for example, by transmitting the contents, or in other words, the signal for tumor growth and progression from tumor cells in other tissues to the brain (97, 98). The use of exosomes in the treatment of brain tumors could be in two ways: 1) Identifying, targeting, and inhibiting exosomes involved in the progression of brain tumors. 2) Treatment of brain tumors by exosomes carrying effective contents such as mRNAs, proteins, and lipids. Although not routinely used today and treatments such as whole-brain radiation therapy, surgery, stereotactic radiosurgery, chemotherapy, anticonvulsant drugs, and steroids are the main guidelines for treating patients with brain tumors (99).

The most prominent feature of exosomes, their size, can be used to indicate the applicability of exosomes for the treatment of brain tumors. The nanometer size of exosomes could be a good tool for crossing the BBB and delivering effective contents. However, further studies are needed to properly understand the potential role of the exosome in treatment protocols. Also, the exosome has been shown to be a safe way to increase the stability of its contents. Therefore, in addition to the ability of the exosome to cross the BBB, the use of the exosome as a suitable coating in protecting the useful contents for the treatment of brain tumors and their delivery can be effective (100).

## Author Contributions

ZH and SK prepared the backbone of the manuscript. ZH, SK, and MI wrote the original draft of the manuscript. Z-SC and MS refined the manuscript. Z-SC critically revised the manuscript. ZH and Z-SC supported the project. All authors approved the submitted version.

## Conflict of Interest

The authors declare that the research was conducted in the absence of any commercial or financial relationships that could be construed as a potential conflict of interest.

## Publisher’s Note

All claims expressed in this article are solely those of the authors and do not necessarily represent those of their affiliated organizations, or those of the publisher, the editors and the reviewers. Any product that may be evaluated in this article, or claim that may be made by its manufacturer, is not guaranteed or endorsed by the publisher.
